# Update on Thromboembolic Events After Vaccination Against COVID-19

**DOI:** 10.3390/vaccines13080833

**Published:** 2025-08-05

**Authors:** Theocharis Anastasiou, Elias Sanidas, Thekla Lytra, Georgios Mimikos, Helen Gogas, Marina Mantzourani

**Affiliations:** 1 Department of Cardiology, “Laiko” General Hospital, 11527 Athens, Greece; easanidas@yahoo.gr; 2 Third Cardiology Department, Sotiria Hospital, National and Kapodistrian University of Athens Medical School, 11527 Athens, Greece; theklalytra@hotmail.com; 3 Division of Genetics & Biotechnology, Department of Biology, National and Kapodistrian University of Athens, 11527 Athens, Greece; mimikos.georg@gmail.com; 4 First Department of Internal Medicine, “Laiko” General Hospital, National and Kapodistrian University of Athens Medical School, 11527 Athens, Greece; hgogas@hol.gr (H.G.); mantzourani@gmail.com (M.M.)

**Keywords:** COVID-19 vaccines, thrombosis, vaccine safety

## Abstract

The association between COVID-19 vaccination and thromboembolic events has garnered significant research attention, particularly with the advent of vaccines based on adenoviral vectors, including AstraZeneca’s and Johnson & Johnson’s vaccines. This review underscores the uncommon occurrence of venous thromboembolism (VTE), arterial thromboembolism (ATE), and vaccine-induced thrombotic thrombocytopenia (VITT) following COVID-19 vaccination. Although these complications are extremely rare compared to the heightened risk of thrombosis from COVID-19 infection, elements like age, biological sex, type of vaccine and underlying health conditions may contribute to their development. In addition, rare renal complications such as acute kidney injury and thrombotic microangiopathy have been documented, broadening the spectrum of potential vaccine-associated thrombotic manifestations. Current guidelines emphasize early detection, individualized risk assessment, and use of anticoagulation therapy to mitigate risks. Despite these events, the overwhelming majority of evidence supports the continued use of COVID-19 vaccines, given their proven efficacy in reducing severe illness and mortality. In addition, recent comparative data confirm that mRNA-based vaccines are associated with a significantly lower risk of serious thrombotic events compared to adenoviral vector platforms. Ongoing research is essential to further refine preventive and therapeutic strategies, particularly for at-risk populations.

## 1. Introduction

The COVID-19 pandemic has been one of the greatest public health crises of this century. The creation of highly efficacious vaccines has provided hope in terms of reducing the pandemic’s effects. However, thromboembolic events following COVID-19 vaccination, although rare, have raised concerns. The occurrence of venous thromboembolism (VTE), arterial thromboembolism (ATE), and vaccine-induced thrombotic thrombocytopenia (VITT) has been reported in some cases [1,2]. Risk factors such as age, sex and pre-existing conditions (e.g., thrombophilia) may increase susceptibility [3,4]. Current guidelines emphasize early detection, appropriate anticoagulation, and vigilant monitoring in high-risk individuals [5]. This review provides an updated analysis of the incidence of and risk factors for post-vaccination thromboembolic events, and the strategies for their prevention and management, contributing to the development of next-generation vaccines.

## 2. Materials and Methods

References for this article were collected through structured searches conducted in PubMed, Google Scholar, the WHO International Clinical Trials Registry Platform (ICTRP), and the websites of regulatory agencies and pharmaceutical companies. The literature search was updated until July 2025. The search terms included combinations of “COVID-19 vaccination”, “thromboembolic events”, “thrombocytopenia”, “hemorrhagic events”, “ChAdOx1-S”, “Ad26.COV2.S”, “BNT162b2”, “mRNA-1273”, “systematic review”, and “meta-analysis”. This search approach ensured the inclusion of studies involving both adenoviral vector-based (e.g., ChAdOx1-S, Ad26.COV2.S) and mRNA-based (e.g., BNT162b2, mRNA-1273) vaccines, thereby enhancing the comprehensiveness and representativeness of the review.

The inclusion criteria focused on peer-reviewed articles, large-scale cohort studies, randomized controlled trials, systematic reviews, and meta-analyses published between 2021 and 2025 that evaluated thromboembolic complications following COVID-19 vaccination. Studies addressing both venous (VTE) and arterial (ATE) thromboembolic events, as well as vaccine-induced thrombotic thrombocytopenia (VITT), were prioritized.

Editorials, preprints, non-peer-reviewed sources, and studies without quantitative outcome data were excluded. Ultimately, 42 high-relevance studies were selected to provide a representative and up-to-date synthesis of the available evidence.

## 3. Results

To enhance the clarity and interpretability of the findings, this section is structured based on the vaccine platform (adenoviral vector-based and mRNA-based vaccines) and type of supporting evidence. Specifically, data are grouped according to the study design—observational cohort studies, randomized controlled trials, pharmacovigilance reports, case series, and systematic reviews—to ensure a logical flow and avoid repetition. This approach facilitates comparison of the thromboembolic outcomes across vaccine types and strengthens the validity of the synthesized conclusions.

### 3.1. Incidence of Post-Vaccination Thromboembolic Events by Vaccine Type

The currently authorized COVID-19 vaccines include four main types: mRNA-based vaccines—BNT162b2 (Pfizer–BioNTech) and mRNA-1273 (Moderna)—and adenoviral vector-based vaccines—ChAdOx1-S (Oxford–AstraZeneca) and Ad26.COV2.S (Janssen/Johnson & Johnson). These vaccines differ in their immunogenic platforms, with mRNA vaccines encoding spike protein mRNA, while adenoviral vectors deliver spike protein DNA via modified viruses. This foundational distinction may partly explain the differing incidence rates of post-vaccination thromboembolic events across vaccine platforms.

**Adenoviral Vector-Based Vaccines**

After millions of doses were administered, rare instances of thrombosis with thrombocytopenia syndrome (TTS) emerged, particularly following the first dose of adenoviral vaccines such as ChAdOx1-S and Ad26.COV2.S [6,7]. In the US, the VAERS estimated the incidence of TTS to be 3.83 cases per million doses for Ad26.COV2.S [8]. A study from Denmark and Norway indicated a small increase in specific thrombotic events following ChAdOx1-S [9], and Scottish data also indicated modest increases in thromboembolic complications with this vaccine [10]. Furthermore, a global study found a 30% higher likelihood of thrombocytopenia after ChAdOx1-S compared to BNT162b2 [11].

In terms of cerebral venous sinus thrombosis (CVST), Kerr et al. reported 16.34 cases per million doses of ChAdOx1-S, while a pooled analysis from the UK found a rate ratio of 1.93 (95% CI: 1.20–3.11) within 28 days of vaccination [12].

**mRNA-Based Vaccines**

The same Scottish study investigating BNT162b2 reported a much lower rate of thromboembolic events compared to adenoviral vector vaccines, supporting a favorable safety profile [10]. VAERS data indicated 0.00855 cases per million doses for TTS associated with mRNA vaccines [8]. Kerr et al. also reported 12.60 cases per million doses of CVST for BNT162b2, lower than for ChAdOx1-S [12].

**Comparative and Pooled Data**

Whiteley et al. observed that arterial and venous thromboembolism were reduced in vaccinated individuals with either ChAdOx1-S or BNT162b2 compared to unvaccinated persons, as shown in a cohort of 46 million people in England [13]. A systematic review and meta-analysis revealed no significant increase in the thromboembolic risk among vaccinated individuals, with a pooled risk ratio of 1.14 (95% CI: 0.61–2.14). While this point estimate could suggest a possible small increase, the wide confidence interval includes the possibility of no effect. Some extrapolations estimate approximately 7.8 additional events per 100,000 individuals, though this value is not definitive and should be interpreted with caution [14].

Another analysis including both mRNA and adenoviral vaccines emphasized that while thromboembolic events are serious, the overall incidence remains low, and the benefits outweigh the risks [15]. An international study reported a frequency of 0.21 cases of thrombotic events per million vaccinated person-days, compared to a 14.7% incidence of VTE following COVID-19 infection [16].

Although most thromboembolic and hemorrhagic events occur within the first few weeks following vaccination, rare delayed complications have also been documented. A recent case report described fatal pulmonary hemorrhage occurring 555 days after the second dose of the BNT162b2 (Pfizer–BioNTech) vaccine, raising awareness of the potential for long-latency adverse vascular outcomes [9]. Additionally, some cohort-based studies have reported isolated cases of pulmonary embolism and venous thromboembolism occurring more than 90 days post-vaccination, particularly in individuals with autoimmune predisposition or pre-existing thrombotic disorders [17]. These findings underscore the necessity of extended pharmacovigilance systems and long-term safety monitoring of vaccinated populations.

A summary of the incidence data stratified by vaccine platform and study design is provided in Table 1.

### 3.2. Adverse Factors for the Occurrence of Post-Vaccination Thromboembolic Events

Numerous sources provide a comprehensive analysis of the factors related to thromboembolic events following COVID-19 vaccination, including individual demographics, vaccine variety and underlying health conditions. This review highlights the significance of interactions between vaccine type and underlying health factors in influencing the risk of events such as VTE and ATE. A higher risk of thrombocytopenia was observed in people in the 40–49 age group, the 70–79 age group and females in the UK who were administered the initial dose of the ChAdOx1-S vaccine, in comparison to those who received the first dose of BNT162b2. Additionally, the rate ratio for arterial thromboembolism following ChAdOx1-S versus BNT162b2 was lower in males, with an adjusted rate ratio of 0.75 (95% confidence interval, 0.61 to 0.92). Subgroup analyses indicated a 25% lower risk of arterial thromboembolism in men who received the initial dose of ChAdOx1-S compared to BNT162b2 in the UK. In contrast, younger individuals (aged 20–29 years) in the US who were vaccinated with Ad26.COV2.S experienced a four to five times higher risk of arterial thromboembolism compared to those who received mRNA vaccines. However, these findings were not consistently observed across other datasets or age groups, indicating the need for further research [11].

A large hospital-based linkage study in England also highlighted a higher risk of CVST following the initial dose of the ChAdOx1-S (AstraZeneca) vaccine, but this risk was confined to adults under 65 years of age. Notably, no such risk was observed after receiving the BNT162b2 (Pfizer) vaccine [18].

A cohort study evaluated various risk factors for adverse effects post-vaccination, including age, sex, pre-existing conditions, and prior COVID-19 infection. This study found that younger individuals, women, and those with had previous COVID-19 infections were more likely to experience negative events, including thromboembolic episodes, though these occurrences were still rare. Among 19,586 participants, the most significant factors associated with adverse effects included vaccine administration, vaccine manufacturer, age group, female gender, and history of COVID-19 infection. Side effects occurred more often after full vaccination with mRNA-1273 and were more prevalent among younger individuals, females, those with a history of COVID-19, those with Asian ethnicity, pregnant women, and marijuana users. In contrast, older age, Black or African American ethnicity, and other factors were linked to a lower likelihood of reporting side effects [28].

Another study identified risk factors for thromboembolic events, showing that while the rates of thrombocytopenia and VTE were generally low after booster doses of both ChAdOx1 and BNT162b2, the risk of VTE was higher in females after the first dose of ChAdOx1, although the increase was not statistically significant. Similar patterns were observed following SARS-CoV-2 infection, with no significant variation across subgroups [22].

This evidence suggests that while underlying health conditions, such as heart disease, obesity, as well as thrombophilia, may increase the likelihood of thromboembolic events after receiving the COVID-19 vaccination, the available information does not suggest a notable rise in the risk after immunization. For instance, among 25,296 people with a history of VTE, the chance of developing VTE after vaccination was lower compared to the period before vaccination (hazard ratio [HR] of 0.77, with a 95% confidence interval between 0.62 and 0.95). Similarly, no significant increase in the VTE risk was noted in 385 patients with a previous occurrence of heparin-induced thrombocytopenia (HIT), and multivariable Cox models showed no notable changes in the likelihood of VTE between the Pfizer, Moderna, and Janssen vaccines. These findings suggest that while pre-existing conditions may influence the VTE risk, there is no consistent evidence of an elevated post-vaccination risk, reinforcing the overall safety of COVID-19 vaccines [24].

Further subgroup analyses from population-based studies have revealed differential risks of thromboembolic events according to the vaccine platform. For instance, adenoviral vector vaccines such as ChAdOx1-S have been associated with a higher incidence of VITT, especially among females under the age of 50, while mRNA-based vaccines (BNT162b2, mRNA-1273) demonstrate a substantially lower risk profile for such complications [11,12]. Age-stratified data indicate that younger adults (18–49 years), particularly females, are at higher risk of thrombocytopenic events after the first dose of adenoviral vaccines, whereas older adults (>65 years) show no increased thrombotic risk. Similarly, individuals with pre-existing prothrombotic conditions or autoimmune disorders have shown slightly higher rates of post-vaccine thromboembolic events, though these remain rare overall [11,24].

According to a case series, thrombosis with thrombocytopenia syndrome is an uncommon yet severe complication linked to Ad26.COV2.S vaccination. The typical age of those affected was 44.5 years (ages ranging from 18 to 70), with 69% of cases occurring in women [8]. Another study reported a significantly elevated risk of venous thromboembolism in expectant mothers during the final trimester of pregnancy, with a 22 times higher risk in the first six weeks postpartum [29].

In one case series, the incidence of vaccine-related immune thrombocytopenic thromboembolism was reported to be less than 1 in 100,000 doses for most COVID-19 vaccines, with a higher risk observed in younger populations, particularly females [30].

Furthermore, a cohort study based on population data carried out in England by Whiteley et al. found a higher incidence of thrombocytopenia within 28 days following the administration of the ChAdOx1-S vaccine compared to unvaccinated individuals under the age of 70. However, no similar association was found with the BNT162b2 vaccine [13].

In summary, these studies reinforce the idea that while certain demographic groups and pre-existing conditions may influence the likelihood of thromboembolic events following vaccination, the overall risk remains low and is outweighed by the advantages of COVID-19 immunization.

### 3.3. Guidelines for the Prevention and Management of Post-Vaccination Thromboembolic Events

Guidelines for managing thromboembolic events after COVID-19 vaccination generally focus on identifying and treating rare conditions like TTS. These guidelines vary depending on the vaccine type and patient risk. Most emphasize early recognition, risk stratification, and individualized treatment.

The CDC issued updated recommendations in April 2021 concerning the Janssen vaccine after TTS reports, especially among women under 50, recommending alternative vaccines for this group while affirming the overall vaccine benefits [5]. Similarly, the Hellenic National Public Health Organization (EODY) recommends monitoring for thrombotic symptoms and use of non-heparin anticoagulants with IV immunoglobulin in suspected VITT cases [25]. Barnes et al. outlined thromboprophylaxis guidelines for hospitalized and non-hospitalized COVID-19 patients, recommending LMWH for inpatients and case-by-case anticoagulation for high-risk outpatients [31]. The WHO advocates LMWH or unfractionated heparin for hospitalized patients and emphasizes individualized care and ongoing research [26].

To conclude, while thromboembolic complications post-vaccination are rare, adherence to evolving international and national guidelines ensures early detection and optimized treatment, thereby enhancing patient safety without undermining vaccine confidence.

### 3.4. Renal Complications Following COVID-19 Vaccination

Emerging evidence highlights that thromboembolic events post-COVID-19 vaccination may extend beyond venous and arterial systems, potentially impacting renal microvasculature and function. Acute kidney injury (AKI), renal thrombosis, and thrombotic microangiopathy (TMA) have been sporadically reported in association with various COVID-19 vaccines, particularly in individuals with predisposing risk factors [32].

Case reports and pharmacovigilance data describe the onset of AKI within days to weeks following both mRNA-based and adenoviral vector-based vaccines [33,34]. The mechanisms are presumed to involve endothelial injury, complement activation, and prothrombotic states triggered by aberrant immune responses. In a cohort-based French pharmacovigilance analysis, 48 cases of post-vaccination AKI were reported within 21 days after vaccination, most frequently following the use of mRNA vaccines (especially mRNA-1273), although the causality remains unconfirmed [35].

Thrombotic microangiopathy (TMA), a syndrome marked by thrombocytopenia, microangiopathic hemolytic anemia, and organ ischemia, has also been described in the context of COVID-19 vaccines. TMA episodes, including vaccine-associated atypical hemolytic uremic syndrome (aHUS), were primarily reported in genetically susceptible individuals. In such patients, vaccination is considered a potential trigger rather than a direct cause [36,37]. A case series from Italy documented TMA onset within 10 days post-BNT162b2 or -ChAdOx1-S vaccination in four patients with complement regulatory gene mutations [37].

Furthermore, a handful of reports concern vaccine-associated renal vein thrombosis, particularly in patients with nephrotic syndrome or prior thrombotic history [27]. Although rare, these cases underline the need for vigilance in high-risk populations.

Emerging hypotheses on the pathophysiology of vaccine-associated thromboembolic events include molecular mimicry between vaccine components and endogenous proteins, immune complex formation involving platelet factor 4 (PF4), complement activation, and endothelial dysfunction induced by spike protein expression. These mechanisms are particularly relevant in rare syndromes such as VITT, where anti-PF4 antibodies mimic heparin-induced thrombocytopenia pathways, even in the absence of prior heparin exposure.

Despite these isolated events, population-level data have not demonstrated a statistically significant increase in renal thrombotic complications attributable to vaccination. The incidence of AKI or TMA remains far lower post-vaccination than among patients hospitalized with severe COVID-19, where direct viral cytotoxicity, cytokine storms, and coagulopathy contribute to multiorgan failure, including renal damage [38].

In summary, while rare renal complications such as AKI, TMA, or renal vein thrombosis have been reported following COVID-19 vaccination, the absolute risk appears minimal. However, clinicians should maintain a high index of suspicion in individuals presenting with hematuria, proteinuria, or renal dysfunction post-immunization, especially those with prior thrombotic or complement-mediated disease. Further surveillance and mechanistic studies are needed to clarify causality and guide risk stratification.

## 4. Discussion

Thromboembolic complications following COVID-19 vaccination represent a multifactorial phenomenon arising from interactions between vaccine components, individual susceptibility factors, and immune responses. While adenoviral vector-based vaccines such as ChAdOx1-S and Ad26.COV2.S have been linked to rare cases of vaccine-induced thrombotic thrombocytopenia (VITT) [6,8,11], more recent large-scale cohort studies published in 2024–2025 further clarify the risk profile [35,36,37]. Notably, analyses from the United States, Europe, and Asia consistently report that the absolute incidence of thromboembolic events remains low, particularly when compared with the prothrombotic state induced by acute SARS-CoV-2 infection [23,24].

The immunopathophysiology of VITT is increasingly understood to involve platelet-activating antibodies against platelet factor 4 (PF4), mimicking mechanisms observed in heparin-induced thrombocytopenia (HIT). However, VITT typically occurs in the absence of heparin exposure and has a unique temporal association with adenoviral vector-based vaccines. This reinforces the importance of distinguishing VITT from other thrombotic syndromes in clinical practice [6].

Recent data also emphasize the transient nature of risk, predominantly following the first dose of adenoviral vaccines, with no sustained long-term increase in thrombotic events [11,39]. Furthermore, updated risk–benefit models reinforce that COVID-19 vaccination prevents far more thromboembolic complications—indirectly by preventing severe COVID-19—than it may rarely induce [39]. Nevertheless, heterogeneity across studies persists, especially regarding cerebral venous sinus thrombosis (CVST) and thrombocytopenia, highlighting the need for nuanced risk stratification [10,11,12].

Importantly, the incidence of thromboembolic events varies across age, sex, and comorbidity profiles. Younger women appear at slightly elevated risk of vaccine-associated thrombosis, while older adults benefit most significantly from immunization in terms of thrombotic risk reduction. Data from real-world pharmacovigilance systems have revealed subtle signals, but these must be interpreted in light of passive surveillance limitations, including underreporting and confounding by indication [13,23]. Moreover, autoimmune diseases, cancer, obesity, and hormonal therapies—including oral contraceptives—have been investigated as modifiers of the VITT risk, although definitive causal relationships remain unproven [11]. Stratified analyses of Scandinavian and UK data suggest multifactorial contributions to susceptibility, warranting individualized vaccine risk assessments in select populations [11,13].

Comparative analyses between mRNA-based vaccines (BNT162b2, mRNA-1273) and adenoviral vector-based vaccines (ChAdOx1-S, Ad26.COV2.S) suggest that mRNA platforms are associated with a lower risk of serious thrombotic events such as VITT and CVST [10,11,12]. Nonetheless, even within adenoviral platforms, the absolute event rates remain markedly low and continue to decline with enhanced screening protocols, clinician awareness, and patient education [11,40].

Further evidence from pharmacovigilance systems and large-scale registry studies published between 2023 and 2025 has provided greater granularity in understanding the epidemiology of VITT. These studies consistently show that VITT primarily affects females under the age of 60 following the first dose of adenoviral vector vaccines [11,40]. Underlying factors such as obesity, estrogen therapy, autoimmune disease, and a prior history of thrombosis may further elevate individual risk profiles [11,18]. Importantly, recent cohort studies (e.g., from the UK, Denmark, and Norway) stratify the outcomes by age, sex, and comorbidity status, confirming that mRNA vaccines are associated with a markedly lower incidence of both venous and arterial thromboembolic complications across all the subgroups [11,13].

Additionally, mechanistic investigations into VITT have identified the central role of anti-PF4 antibodies in the absence of heparin exposure, suggesting a distinct immune-mediated prothrombotic state [6,8]. This has led to clinical recommendations for prompt diagnosis and use of non-heparin anticoagulants and IVIG, particularly in younger women presenting with thrombocytopenia and thrombosis after adenoviral vaccination [25,26].

Interestingly, emerging evidence suggests a lower-than-anticipated risk of pulmonary embolism (PE) or deep vein thrombosis (DVT) after mRNA vaccines, as well as possible overestimations in earlier pharmacovigilance reports due to reporting bias [41]. Despite these reassuring findings, vigilance remains essential, particularly in younger women and individuals with underlying thrombophilia or prior venous thromboembolism history [4,24]. Although the vast majority of vaccine-associated thromboembolic events occur within the first 4–30 days following immunization, an increasing number of case reports and registry analyses have drawn attention to the potential for delayed complications. Notably, AlShareef et al. described a rare but fatal case of pulmonary hemorrhage occurring 555 days after administration of a second dose of the BNT162b2 mRNA vaccine, raising concerns about the possibility of very-late-onset immune-mediated vascular injury even in recipients of mRNA platforms [9]. In addition, population-level data and cohort analyses have identified sporadic cases of venous thromboembolism (VTE) and pulmonary embolism (PE) arising beyond 90 days post-vaccination, particularly among individuals with autoimmune disease, genetic thrombophilia, or other predisposing conditions [3,17]. Although these events are exceedingly rare and causality remains difficult to establish, they emphasize the necessity of extended pharmacovigilance and long-term follow-up within vaccine safety registries to capture the full temporal spectrum of the thrombotic risk.

In addition to the well-recognized cardiovascular and neurological complications, thromboembolic events following COVID-19 vaccination may also affect the renal vasculature, albeit rarely. Emerging case reports and cohort analyses have described instances of acute kidney injury (AKI), renal artery thrombosis, and thrombotic microangiopathy (TMA), particularly in temporal association with adenoviral vector-based vaccines [32,33,37]. For instance, biopsy-proven cases of post-vaccination TMA have been reported in patients who developed AKI, proteinuria, and hematuria within days of ChAdOx1-S or Ad26.COV2.S administration [37]. Histological patterns resembling atypical hemolytic uremic syndrome (aHUS) have raised concern about vaccine-triggered complement activation in genetically predisposed individuals [36,37]. Furthermore, sporadic cases of renal infarction and renal vein thrombosis have been documented, particularly among patients with underlying prothrombotic risk factors such as lupus or antiphospholipid syndrome [27]. While causality remains difficult to establish, these observations reinforce the importance of renal function monitoring in the early post-vaccination period for patients with known glomerular diseases, autoimmune disorders, or unexplained thrombocytopenia [32,34]. Given the shared pathophysiological mechanisms of endothelial injury, complement dysregulation, and platelet activation, future mechanistic studies are warranted to delineate the immunothrombotic pathways linking COVID-19 vaccination and renal vascular injury [36,38].

In clinical practice, the emphasis remains on early recognition, individualized risk assessment, and prompt management of thrombotic complications. Updated clinical guidelines advocate for rapid initiation of non-heparin anticoagulants and intravenous immunoglobulin in cases suggestive of VITT, especially within 4–30 days following adenoviral vector vaccine administration [25,26]. Clinicians should also consider baseline risk assessment in patients with known prothrombotic disorders or personal history of thrombosis prior to vaccination [4,24]. Furthermore, international public health agencies—including the CDC, EMA, and WHO—have revised their guidance in light of cumulative data [5,25,26]. These updates stress not only the rare nature of vaccine-associated thrombosis but also the imperative to maintain public confidence in vaccination programs. Transparent communication of risks, coupled with clear benefit–risk comparisons, has been key to sustaining vaccine uptake [39]. However, further studies are warranted to elucidate the immunopathogenesis of VITT and refine prevention strategies in high-risk populations [28,40]. Prospective registries, harmonized definitions of vaccine-induced adverse events, and post-marketing surveillance are crucial components of ongoing safety evaluations [8,26,30]. Additionally, mechanistic research exploring the role of adenoviral vectors, host immunogenetics, and PF4–vaccine interactions will be essential to inform next-generation vaccine design and safety optimization [6,26,36].

Future vaccination strategies may benefit from a stratified approach, whereby adenoviral vector-based vaccines are selectively administered to populations with the most favorable benefit–risk profile [39,40]. Tailored public health communication should also address demographic-specific concerns, particularly in younger females and individuals with predisposing thrombotic risk factors, to preserve public confidence and ensure equitable vaccine uptake.

Although the majority of thromboembolic complications occur within the initial 4–30 days following vaccination, accumulating evidence indicates the possibility of delayed vascular events. A striking example includes a case report of fatal pulmonary hemorrhage occurring 555 days after the administration of a second BNT162b2 dose, underscoring the potential—albeit rare—for long-latency immune-mediated vascular complications even in recipients of mRNA vaccines [9]. Complementary population-level data further suggest that venous thromboembolism (VTE) and pulmonary embolism (PE) may occasionally manifest beyond the traditionally observed risk window, particularly in individuals with autoimmune disorders or known thrombophilic predispositions [4,24,41].

These findings collectively emphasize the need for prolonged safety surveillance and longitudinal registry-based monitoring. Conventional pharmacovigilance systems may be inadequate to capture late-arising adverse events due to passive reporting limitations; thus, evolving toward structured, prospective data collection models is imperative [17,28,35].

In conclusion, thromboembolic events following COVID-19 vaccination remain infrequent, temporally confined, and most often associated with adenoviral vector vaccines. A robust body of real-world and registry-based evidence consistently reaffirms the overall safety of COVID-19 immunization programs, especially those utilizing mRNA-based platforms [12,13,28]. Continued monitoring, risk stratification, clinician education, and translational research are essential to ensure the ongoing success of and public trust in vaccination initiatives globally.

## 5. Conclusions

Current evidence suggests that thromboembolic events after receiving the COVID-19 vaccine are uncommon, especially in individuals with pre-existing conditions or those receiving adenoviral vector vaccines. Although these events are infrequent, they can be severe, prompting specific guidelines for prevention and management. The identified contributing factors—like age, gender, vaccine type, and underlying health conditions—help in stratifying individuals who may require closer monitoring post-vaccination. Importantly, the overall data endorses the continued administration of COVID-19 vaccines, as their effectiveness in reducing severe outcomes of COVID-19 far outweighs the risks of thromboembolic complications. Clinicians should remain vigilant for symptoms indicative of VITT or other thrombotic events, ensuring timely diagnosis and appropriate use of anticoagulant therapy. In parallel, emerging evidence also highlights the need for extended surveillance windows and registry-based long-term monitoring to capture delayed thrombotic or renal complications that may fall outside standard reporting time frames. The integration of immunologic and genetic risk stratification into vaccine safety protocols may further improve individualized assessment and early detection of adverse events. Moreover, real-world pharmacovigilance and harmonized global data-sharing will be instrumental in elucidating the true incidence and pathogenesis of these rare but clinically relevant complications. Further research is warranted to refine risk assessment and management strategies for high-risk populations. Future studies should also address the evolving context of booster strategies and variant-adapted vaccines and evaluate whether individuals who have experienced vaccine-related thrombotic events are at long-term risk of recurrence. Continued interdisciplinary collaboration and sustained public health messaging are essential to maintain trust in vaccination efforts and optimize safety in diverse populations.

## 6. Future Directions

Future research should continue to explore the immunological, genetic, and molecular mechanisms underlying vaccine-related thromboembolic complications, particularly in the context of VITT [28,40]. The key unanswered questions include the role of innate immune priming, the formation of platelet-activating antibodies, and the structural mimicry between vaccine components and platelet factor 4 (PF4). Genomic and immunologic profiling may help identify individuals predisposed to exaggerated immune responses against platelet factor 4 (PF4) or other autoantigens [3,40]. Such approaches could enable the development of pre-vaccination screening tools in high-risk individuals, particularly those with known autoimmune conditions or prior thrombotic events Additionally, further risk–benefit analyses stratified by age, sex, ethnicity, and comorbidities will refine individual-level risk prediction [39,41]. More granular pharmacoepidemiological data are needed to define the risk of thromboembolism in specific subpopulations, such as immunocompromised patients, pregnant women, or those receiving heterologous vaccine schedules.

New studies should also address the evolving landscape of booster doses, variant-adapted vaccines and recombinant protein platforms, where the thromboembolic risks appear minimal but remain under investigation [40,41]. The development of next-generation adenoviral vectors or alternative delivery platforms with reduced immunogenicity may further mitigate these rare complications [40]. Design refinements such as codon optimization, capsid modification, or non-viral nanoparticle vectors may enhance immunogenicity while minimizing off-target effects.

Efforts should also focus on standardizing post-vaccination thrombosis management guidelines [5,42] and creating international registries for long-term follow-up of patients who experience vaccine-related thrombotic events, evaluating whether these individuals face an increased lifetime risk of thrombosis [5], including recurrent venous thromboembolism, chronic thromboembolic pulmonary hypertension (CTEPH), or long-term hematologic sequelae. In parallel, harmonization of the adverse event definitions across regulatory agencies will enable more consistent reporting and pooled safety analyses.

Moreover, integration of real-world evidence with controlled trial data will be essential for informing regulatory decisions, particularly as COVID-19 vaccines continue to evolve and be deployed in booster campaigns. Collaboration between pharmacologists, immunologists, hematologists, and public health experts will be necessary to bridge mechanistic insights with clinical applications.

Overall, despite extensive recent research, substantial knowledge gaps remain regarding the immunological triggers, optimal prevention strategies, and long-term vascular outcomes in this rare but clinically significant complication of vaccination [11,39]. Addressing these gaps will also have broader implications for vaccine safety science, especially in the context of emerging infectious diseases and rapidly deployed immunization campaigns. Sustained pharmacovigilance, coupled with interdisciplinary collaboration, will be key to safeguarding vaccine safety and maintaining public trust in immunization efforts [31,42].

## Figures and Tables

**Table 1:** Summary of thromboembolic event incidence by study type and vaccine platform.

*Study Type*	*Adenoviral Vector Vaccines (ChAdOx1-S, Ad26.COV2.S)*	*mRNA Vaccines (BNT162b2, mRNA-1273)*
Randomized Controlled Trials (RCTs)	[5,8,10,12,13,18,19]	[10,12,13,18,20,21]
Observational Cohort Studies	[1,11,13,18,19,22,23]	[1,3,11,13,18,22,23,24]
Pharmacovigilance and Registry Data	[5,8,25,26]	[7,8,25,26]
Case Reports/Case Series	[8,9,12,15]	[9,12,15,27]
Systematic Reviews/Meta-Analyses	[2,14,16]	[2,14,16]

## Data Availability

No new data were created or analyzed in this study. Data sharing is not applicable to this article.

## Abbreviations

The following abbreviations are used in this manuscript:

COVID-19	Coronavirus Disease 2019
VTE	Venous Thromboembolism
ATE	Arterial Thromboembolism
VITT	Vaccine-Induced Thrombotic Thrombocytopenia
TTS	Thrombosis with Thrombocytopenia Syndrome
CVST	Cerebral Venous Sinus Thrombosis
EMA	European Medicines Agency
VAERS	Vaccine Adverse Event Reporting System
CDC	Centers for Disease Control and Prevention
LMWH	Low-Molecular-Weight Heparin
DOACs	Direct Oral Anticoagulants
WHO	World Health Organization
